# Mycobacterium smegmatis Infection in a Cardiovascular Implantable Electronic Device in an Immunocompetent Patient

**DOI:** 10.7759/cureus.115768

**Published:** 2026-09-04

**Authors:** Mustafa Nuaimi, Sara Ubosy, Antonio Crespo, Daniela Tello, Steve Carlan

**Affiliations:** 1 Internal Medicine, Orlando Regional Medical Center, Orlando, USA; 2 Infectious Diseases, Orlando Regional Medical Center, Orlando, USA; 3 Academic Affairs and Research, Orlando Regional Medical Center, Orlando, USA

**Keywords:** cardiovascular implantable electronic device (cied), catheter-associated bacteremia, mycobacterium smegmatis, non-tuberculous mycobacteria (ntb), rapidly growing mycobacteria (rgm)

## Abstract

Cardiovascular implantable electronic devices (CIEDs) are increasingly utilized for arrhythmia management, but device-related infections remain a significant complication. Rapidly growing non-tuberculous mycobacteria (RGM), including *Mycobacterium smegmatis*, are rare causes of CIED infections, typically reported in immunocompromised patients or in association with indwelling catheters. These infections pose diagnostic and therapeutic challenges due to non-specific clinical presentations and intrinsic antimicrobial resistance.

We report an immunocompetent elderly patient who developed complete heart block requiring pacemaker implantation. The patient subsequently experienced device-related complications, including lead dislodgement and multiple revisions, followed by development of a pacemaker pocket site infection. Blood cultures grew *M. smegmatis*, and imaging revealed a significant fluid collection at the pocket site. The patient was managed with complete device removal, placement of a leadless pacemaker, and targeted multidrug antimicrobial therapy guided by susceptibility testing. The patient responded favorably, with resolution of bacteremia and pocket site infection.

This case highlights the importance of considering *M. smegmatis* as a potential pathogen in CIED infections, even in immunocompetent hosts. Successful management requires prompt recognition, source control through device removal, accurate microbiologic diagnosis, and prolonged, susceptibility-guided antimicrobial therapy. Clinicians should maintain a high index of suspicion for RGM infections in patients with indwelling device infections, particularly when initial empiric therapy fails. Although rare, *M. smegmatis* CIED infections can occur in immunocompetent patients and are associated with device manipulation or revisions. This report underscores the need for vigilance, multidisciplinary management, and prolonged follow-up in such complex infections.

## Introduction

Cardiovascular implantable electronic devices (CIEDs), including pacemakers (PMs) and implantable cardioverter-defibrillators (ICDs), are increasingly used worldwide, with implantation rates steadily rising over the past two decades [1]. Although these devices significantly reduce morbidity and mortality in appropriately selected patients, they carry a risk of infection, reported in about 1%-2% of cases [2]. The risk is higher in patients undergoing device revisions or requiring multiple leads. Most CIED infections are caused by Gram-positive bacteria, mainly coagulase-negative staphylococci and *Staphylococcus aureus*, which together account for nearly 70% of cases [3]. In contrast, infections caused by rapidly growing non-tuberculous mycobacteria (RGM) are rare but increasingly recognized in association with implanted medical devices [4]. The most commonly involved species include *Mycobacterium fortuitum* and *Mycobacterium abscessus*, while *Mycobacterium smegmatis* remains an uncommon pathogen [5]. Analysis of the PubMed literature shows that *M. smegmatis* skin and soft tissue infections in adults are rare, with only 21 reported cases, most associated with trauma or surgery. Additional reports describe cases of pneumonia, joint infections, endocarditis, ocular infection, disseminated disease, and at least six cases of catheter-associated bacteremia [6].

Despite these varied presentations, *M. smegmatis* has rarely been linked to CIED infections. Available reports indicate that RGM-related CIED infections pose significant diagnostic and therapeutic challenges because clinical signs may mimic postoperative hematoma or superficial cellulitis [7]. Furthermore, RGM are inherently resistant to many common antimicrobial agents, necessitating prolonged multidrug therapy guided by susceptibility testing [8]. Successful management generally involves complete device removal, targeted antimicrobial therapy, and careful monitoring for complications such as recurrence, bacteremia, or device-related endocarditis.

We report the first known case of catheter-associated bloodstream infection with *M. smegmatis* in an immunocompetent patient undergoing revision of a CIED for complete heart block, occurring about three weeks after device implantation. Prompt recognition and appropriate management, including device removal, tissue sampling, and antimicrobial susceptibility testing, are crucial for successful outcomes in these patients.

## Case presentation

An 82-year-old woman with coronary artery disease and recent percutaneous coronary intervention to the mid-left anterior descending artery presented with a progressively complicated PM pocket infection caused by *M. smegmatis*. Her medical history was notable for coronary artery disease diagnosed approximately five years earlier, treated with percutaneous coronary intervention and stent placement. On admission, examination and imaging ruled out active pulmonary infection. On Day 1, she underwent repeat angioplasty with mid-left anterior descending artery stent placement. Her hospitalization was complicated by complete atrioventricular block requiring temporary transvenous pacing. A permanent PM was subsequently implanted on Day 2; however, right ventricular lead dislodgement necessitated system revision on Day 4.

Approximately three weeks after the revision, she developed progressive swelling and erythema over the left anterior chest PM pocket. She presented to the emergency department on Day 27, and again on Day 29, with worsening pocket swelling and erythema extending onto the adjacent chest wall. She had no significant systemic symptoms and remained hemodynamically stable. Ultrasound demonstrated edema and a small amount of fluid anterior to the PM with possible debris and septations, interpreted primarily as a postoperative hematoma with possible superimposed superficial cellulitis. Inflammatory markers were elevated (C-reactive protein (CRP) 5.11 mg/dL), but she had no leukocytosis and remained essentially afebrile, with a maximum temperature of 37.9°C. Methicillin-resistant *S. aureus* screening was negative. She was treated empirically with doxycycline for seven days, completing therapy on Day 36.

Unexpectedly, two sets of blood cultures obtained on Day 29 grew *M. smegmatis*, with a time to positivity of approximately 3.8 days. The finding was initially challenging to interpret because the patient lacked systemic manifestations of infection and the pocket abnormality had been attributed to a postoperative hematoma. Susceptibility testing was subsequently referred to tertiary laboratory services. She had no reported water exposure to the PM site or other obvious environmental exposure to RGM.

Despite initial conservative management, the local process progressed. On Day 45, she returned to the hospital with one to two days of copious purulent drainage from the PM pocket and new swelling of the ipsilateral left upper extremity. She reported rigors but continued to deny fever. Examination and laboratory evaluation again demonstrated a striking absence of systemic inflammatory findings, with no leukocytosis. Repeat blood cultures obtained on January 2 were negative. Given the progression from a presumed postoperative hematoma to frank purulent discharge in the setting of previously documented *M. smegmatis* bacteremia, a PM pocket infection due to *M. smegmatis* was strongly suspected.

The clinical course was further complicated by acute left upper-extremity venous thrombosis, with venous Doppler ultrasonography demonstrating acute thrombosis involving the left subclavian, axillary, proximal basilic, and median cubital veins. She initially received broad-spectrum antibacterial therapy on admission after consultation with Infectious Disease, including cefepime, vancomycin, trimethoprim-sulfamethoxazole (TMP-SMX), doxycycline, and a single dose of metronidazole. However, because of the prior *M. smegmatis* bacteremia and the presence of a foreign-body infection requiring source control, therapy was redirected toward RGM with amikacin, linezolid, and TMP-SMX based on Infectious Disease consultation.

On Day 46, her labs revealed an abnormal erythrocyte sedimentation rate (ESR), and the entire PM system was removed, and a leadless PM was implanted (Table 1).

**Table 1:** Lab parameters on admission

Parameter	Reference range	Value
Hemoglobin	11.4-14.7 g/dL (grams/deciliter)	14.5
WBC (white blood cell)	4.4-10.5 u/L (units/liter)	6.2
Platelets	139-361 u/L (units/liter)	343
BUN (blood urea nitrogen)	7-20 mg/dL (milligrams/deciliter)	20
Creatinine	0.6/1.1 mg/dL (milligrams/deciliter)	0.98
Potassium	3.5-5.1 mg/dL (milligrams/deciliter)	4.7
ESR (erythrocyte sedimentation rate)	0-30 mm/hr (millimeters/hour)	49
CRP (C-reactive protein)	<1 mg/dL (milligrams/deciliter)	2.08
TSH (thyroid-stimulating hormone)	0.35-4.9 u/L (units/liter)	1.8

Transthoracic echocardiography showed no evidence of valvular or intracardiac vegetations. Intraoperative cultures from the PM pocket and device remained negative, possibly due to antibiotic therapy, despite the previous positive blood cultures and subsequent purulent pocket drainage. She was discharged on intravenous amikacin administered three times weekly in combination with linezolid and TMP-SMX while awaiting formal susceptibility testing.

Approximately three weeks later, susceptibility testing demonstrated that the isolate was susceptible to multiple agents, including cefoxitin, imipenem, fluoroquinolones, doxycycline, TMP-SMX, linezolid, and amikacin, but resistant to clarithromycin. During treatment, the patient developed new hearing loss concerning for aminoglycoside-associated ototoxicity. Amikacin was therefore discontinued on Day 67, and antimicrobial therapy was transitioned to oral doxycycline plus TMP-SMX, with a planned treatment duration of at least six months.

The clinical course was complicated further by recurrent swelling at the prior PM pocket site. At follow-up on Day 77, she reported renewed swelling over the left chest wall despite overall clinical improvement. Ultrasound demonstrated a large complex fluid collection measuring 10.5 × 9 × 3 cm. On Day 80, ultrasound-guided aspiration yielded 130 mL of reddish-brown complex fluid. Importantly, cultures of the aspirated fluid remained negative, providing no microbiologic evidence of recurrent infection and raising consideration of a persistent postoperative or inflammatory collection rather than uncontrolled mycobacterial infection.

At subsequent follow-up, the patient reported complete resolution of the prior chest wall infection without recurrent discharge, fever, or systemic symptoms. A retained suture at the previous pocket site was removed during a cardiology visit, while another protruding suture subsequently fell out spontaneously, after which local healing continued to improve. Residual hyperpigmentation at the former infection site persisted but without evidence of active infection. She continued doxycycline and TMP-SMX twice daily with only mild gastrointestinal intolerance, which improved when the medications were taken with meals and adequate water.

## Discussion

*M. smegmatis* is an RGM increasingly recognized as an opportunistic pathogen. A review of the published literature identified 21 cases of *M. smegmatis* skin and soft tissue infections, typically associated with trauma or surgery [9-14]. Clinical presentations have included post-traumatic ulcerated nodules [11], soft-tissue abscesses [12], surgical site infections after cosmetic procedures [13], and hand infections. Additional reports highlight infections in immunocompromised patients [14]. Beyond soft tissue disease, *M. smegmatis* has also been linked to pleuropulmonary infections [6], pediatric lipoid pneumonia [15], and fatal pulmonary disease in infants [16]. Cases of joint infections [9], isolated endocarditis, corneal ulcers following keratotomy, disseminated disease [10], and six cases of catheter-associated bacteremia [9,17] have also been documented.

Catheter-associated infections caused by RGM are well documented, especially with long-term central venous catheters (CVCs), peritoneal dialysis catheters, or shunt devices. The most commonly involved organisms include *M. fortuitum* and *Mycobacterium mucogenicum* [18]. These infections often present with fever, local inflammation, pulmonary infiltrates, or bacteremia, particularly in immunocompromised hosts. Although rare, *M. smegmatis* has been reported to cause CVC-associated bacteremia, mainly in oncology patients [9,17,19].

To our knowledge, this case is the first report of *M. smegmatis* device-associated bloodstream infection in an immunocompetent patient. This observation is relevant to the broader group of cardiovascular infections related to RGM, including CIED infections. The placement of CIEDs-including PMs and ICDs-has increased significantly. RGM, chiefly *M. fortuitum* and *M. abscessus*, have been reported, accounting for nearly 50% of atypical mycobacterial CIED infections [5]. Additional cardiovascular manifestations of RGM include sternal wound infections [12] and endocarditis following cardiac surgery. The pathogenicity of RGM is linked to their environmental ubiquity and rapid growth potential, with mature colonies appearing within seven days. Taxonomically, clinically relevant RGM can be categorized into three major groups: the *M. fortuitum* complex, the *M. chelonae-abscessus* complex, and the *M. smegmatis* group [13]. *M. smegmatis* remains a rare pathogen compared to other RGM, making recognition particularly challenging. Diagnosis requires a high index of suspicion, especially in catheter- or device-associated infections, where routine bacterial cultures may be negative. The most recent American Heart Association (AHA) statement recommends that both lead tips and tissue be cultured for fungi and mycobacteria if Gram stains are unrevealing, and that mycobacterial stains be performed on resected pocket tissue [10]. In our case, blood cultures and cultures from the removed device material confirmed *M. smegmatis*, despite negative acid-fast bacilli stains on wound and tissue specimens. Treatment of RGM infections is challenging due to variable susceptibility profiles and the lack of standardized guidelines for device-associated infections. *M. smegmatis* is typically susceptible to sulfonamides, doxycycline, imipenem, and amikacin but resistant to cephalosporins and macrolides [19]. Current American Thoracic Society/Infectious Diseases Society of America (ATS/IDSA) guidelines recommend at least two active agents for 4-6 months, combined with removal of infected devices [17]. Therapy must be individualized and guided by susceptibility testing, as illustrated in our case, where initial broad therapy was later narrowed to doxycycline and TMP-SMX following confirmatory testing. Importantly, monitoring for drug-related toxicities (e.g., QTc prolongation with macrolides or quinolones) is essential, particularly in patients with underlying cardiac disease. This case adds to the limited literature by demonstrating that *M. smegmatis* can cause CVC-associated bacteremia even in immunocompetent hosts. Clinicians should consider RGM in the differential diagnosis of catheter- and device-associated infections, regardless of host immune status. Prompt source control, tailored antimicrobial therapy, and close monitoring remain the cornerstone of management. The clinical relevance of this case is likely greatest in patients with persistent pocket-site abnormalities, repeated device manipulation or revision, bloodstream involvement, or microbiologic findings suggestive of RGM. These observations should not be generalized to routine uncomplicated CIED infections.

Overall, this case illustrates an indolent but destructive *M. smegmatis* cardiac implantable electronic device infection that initially masqueraded as a postoperative hematoma and superficial cellulitis. The absence of fever, leukocytosis, or echocardiographic evidence of endocarditis did not exclude clinically significant device infection. The combination of persistent pocket inflammation, subsequent purulent drainage, and positive blood cultures for a RGM ultimately necessitated complete device removal and prolonged targeted antimicrobial therapy. The case also highlights the therapeutic challenge of balancing prolonged antimycobacterial treatment against drug toxicity, as demonstrated by amikacin-associated hearing loss, and the importance of distinguishing recurrent infection from sterile postoperative fluid collections during follow-up.

This case also highlights the importance of considering RGM such as *M. smegmatis* in selected patients with persistent or atypical device-related infections, particularly after device manipulation or revision. Although conclusions from a single case report are inherently limited, our findings suggest that delayed clinical improvement, negative routine cultures, or recurrent pocket-site abnormalities may warrant expanded microbiologic evaluation and susceptibility-guided therapy. Broader management implications should be interpreted cautiously and in the context of existing literature.

## Conclusions

This case describes a rare M. smegmatis CIED infection with bloodstream involvement in an immunocompetent patient following device revision. The report emphasizes the importance of considering RGM in selected patients with persistent or atypical post-procedural device-site inflammation, particularly when routine evaluation is unrevealing. Device removal, appropriate specimen collection, and susceptibility-guided antimicrobial therapy were important components of management. Given the descriptive nature of a single case report, these findings should be interpreted as hypothesis-generating clinical observations rather than broadly generalizable recommendations.
